# Flexible Composition: MEG Evidence for the Deployment of Basic Combinatorial Linguistic Mechanisms in Response to Task Demands

**DOI:** 10.1371/journal.pone.0073949

**Published:** 2013-09-12

**Authors:** Douglas K. Bemis, Liina Pylkkänen

**Affiliations:** a Department of Psychology, New York University, New York, New York, United States of America; b Department of Linguistics, New York University, New York, New York, United States of America; c NYU Abu Dhabi Institute, New York University Abu Dhabi, Abu Dhabi, United Arab Emirates; University College of London - Institute of Neurology, United Kingdom

## Abstract

The present study investigates whether a minimal manipulation in task demands can induce core linguistic combinatorial mechanisms to extend beyond the bounds of normal grammatical phrases. Using magnetoencephalography, we measured neural activity evoked by the processing of adjective-noun phrases in canonical (*red cup*) and reversed order (*cup red*). During a task not requiring composition (verification against a color blob and shape outline), we observed significant combinatorial activity during canonical phrases only – as indexed by minimum norm source activity localized to the left anterior temporal lobe at 200–250 ms(cf. [1], [2]). When combinatorial task demands were introduced (by simply combining the blob and outline into a single colored shape) we observed significant combinatorial activity during reversed sequences as well. These results demonstrate the first direct evidence that basic linguistic combinatorial mechanisms can be deployed outside of normal grammatical expressions in response to task demands, independent of changes in lexical or attentional factors.

## Introduction

Human language derives its expressive power from the ability to creatively construct complex meanings out of individual pieces. While this creativity is clearly evident when parsing grammatical expressions, as when constructing a meaning for *purple gorillas sing Vivaldi awkwardly*, it is unclear to what degree the combinatory mechanism of language can be applied outside the bounds of normal language processing; a question relevant both for assessing modularity within the language architecture [3], [4] and for determining the interplay between linguistic combinatorial mechanisms and the cognitive sphere more generally [5]. In the present study, we investigate whether a simple shift in task demands, independent of changes in lexical or attentional requirements, is sufficient to provoke the engagement of basic combinatorial linguistic mechanisms – those that sit at the heart of language and compose complex meanings out of individual elements – beyond their natural, grammatical domain. Specifically, we aimed to assess whether the combinatory mechanisms that compose simple phrases such as *red cup* can also operate on expressions not conforming to the native grammar, such as the reversed sequence *cup red*, in situations in which there is some pressure to interpret the sequence in a combinatory fashion. Intuitively, the combinatory mechanism does have this flexibility, given that comprehension of non-native speakers can be quite good even in the presence of many grammatical errors, such as this type of word order reversal. But intuition does not yet tell us whether the mechanism employed to construct complex representations from ungrammatical input is the same as we employ during the processing of grammatical material. In the current study we assessed this by recording magnetoencephalography (MEG) activity during the processing of grammatical and ungrammatical phrases under combinatory and non-combinatory task demands. The resulting spatio-temporal maps of neural activity allowed us to assess whether basic combinatorial neural mechanisms that operate during normal grammatical processing can be deployed to novel contexts in response to a subtle shift in task demands.

### Previous Studies on the Bounds of Combinatory Language Processing

Few previous studies have directly investigated the extent to which combinatorial linguistic mechanisms can be flexibly deployed to novel contexts, though much work touches upon this question indirectly. Clearly, at some level, combinatorial linguistic processes can be applied to novel contexts, as people are capable of learning to extract meaning from written words and foreign languages, neither of which evoke successful combinatorial linguistic processing without instruction. Neurolinguistic investigations into both types of processing, while not definitive, indicate a large overlap in the neural signatures associated with combinatorial speech comprehension and those evoked both by reading [2], [6], [7] and foreign language processing [8], [9], [10]. More relevantly, a large body of evidence suggests that linguistic mechanisms, broadly construed, can be extended to the processing of artificial ‘languages’ as well. In the canonical artificial grammar learning (AGL) paradigm [11], sets of arbitrary symbols, ranging from foreign words [12], to letter strings [13], to visual objects [14], are generated using a finite automaton such that they obey various syntactic constraints. Subjects are then shown exemplars from these sets during a training period and subsequently asked to judge the ‘grammaticality’ of a test set of strings, some of which are generated by the automaton and some of which are not. There are countless variations on the paradigm, in terms of syntactic constraints, ‘language’ symbols, learning method, and more (see [15] for a review), however, a consistent finding from both behavioral [16], [17] and neural studies [18], [12], [13] is that the processing of such artificial grammars can appear similar to that of natural language in many respects. Thus, in broad strokes, AGL studies provide evidence that linguistic mechanisms can be deployed to novel contexts given appropriate task demands.

Nevertheless, several factors prevent these results from directly addressing the concern of the present study, which is to determine the extent to which basic linguistic combinatorial mechanisms can be flexibly and rapidly deployed to novel contexts. First, in AGL paradigms, though subjects are usually able to distinguish ‘grammatical’ strings from ‘ungrammatical’ strings with better than chance accuracy [15], their performance is rarely perfect [19], and in some circumstances the underlying grammatical rules cannot be mastered at all, despite their apparent similarity to natural language constraints [20], [21]. Second, by design these studies almost exclusively measure processing associated with complex rules, such as hierarchal, nested structures [22], [14] and non-rigid distance dependencies [23], and often assess neural activity generated by the violation of these rules (e.g. [13]). Thus, notorious difficulties in disentangling the myriad of mechanisms that underlie such complex processing in natural language [24], [25] are exaggerated when dealing with artificial languages (cf. [26]). Consequently, there has been extensive disagreement as to the nature of the neural mechanisms that drive effects observed in AGL paradigms [27], [28], [29], [30], [23] or even whether the putative rules have been learned at all [20]. Thus, while results from AGL paradigms suggest an ability to deploy linguistic mechanisms to novel contexts, their relationship to basic linguistic combinatorial processes on the one hand and expectation violation [30], recursion [19], and hierarchical sequencing mechanisms on the other [14], [27] has not yet been entirely resolved.

### Task Manipulations

Within paradigms more straightforwardly directed at investigating the interaction between task demands and linguistic mechanisms, the primary focus has been to determine the effect of attentional manipulations on language processing. In these studies attention is often directed away from the linguistic stimuli altogether, either passively, such as watching a silent movie while listening to speech [31], [32], or actively, such as performing an auditory discrimination task while listening to speech [33] Alternatively, attention can be directed towards or away from different aspects of the stimuli, such as by performing a font detection task [34] or selectively monitoring for syntactic or semantic violations [35]. Roughly, these investigations have uncovered a gradated effect of attention on processing such that early processing stages appear to be largely invariant under attentional manipulations [36], [37], [38], [33], mid-stage processing can often be modulated but not usually eliminated entirely [39], [40], [41], and later processing can come and go depending on the task [34], [42], [37], [43], [44] for a review.

This latter result, of course, potentially suggests the flexible deployment of linguistic mechanisms. Recent work has indeed indicated that later stages of linguistic parsing, often related to syntactic reanalysis and ambiguity resolution, are task dependent to some degree and do not always occur during normal language processing [45], [46], [47], [48]. These results, however, have been primarily attributed to the depth of processing of the linguistic stimuli and have consequently fallen under the heading of ‘good enough’ parsing – i.e. the evidence indicates that during normal language comprehension processing need only be ‘good enough’ to solve the task at hand. Neurophysiologically, a common measure of this processing, the P600 ERP component, is heavily influenced by whether or not a judgment of plausibility is demanded by the given task (see [44]). Thus, rather than reflect the flexible deployment of processes outside of their natural context, these results seem to indicate that parsing may halt before fully engaging mechanisms related to ambiguity resolution or syntactic reanalysis [47]. To date, no evidence has directly addressed whether linguistic combinatorial mechanisms only operate on grammatical input, excepting perhaps work on grammatical illusions in which the parser becomes confused by expressions that appear grammatical despite being representationally ill-formed [49]. Thus, rather than suggesting the flexible engagement of linguistic processes outside of their normal bounds, results related to ‘good enough’ parsing speak more to their automaticity within the normal grammatical domain.

### Our Study

**Figure 1:**
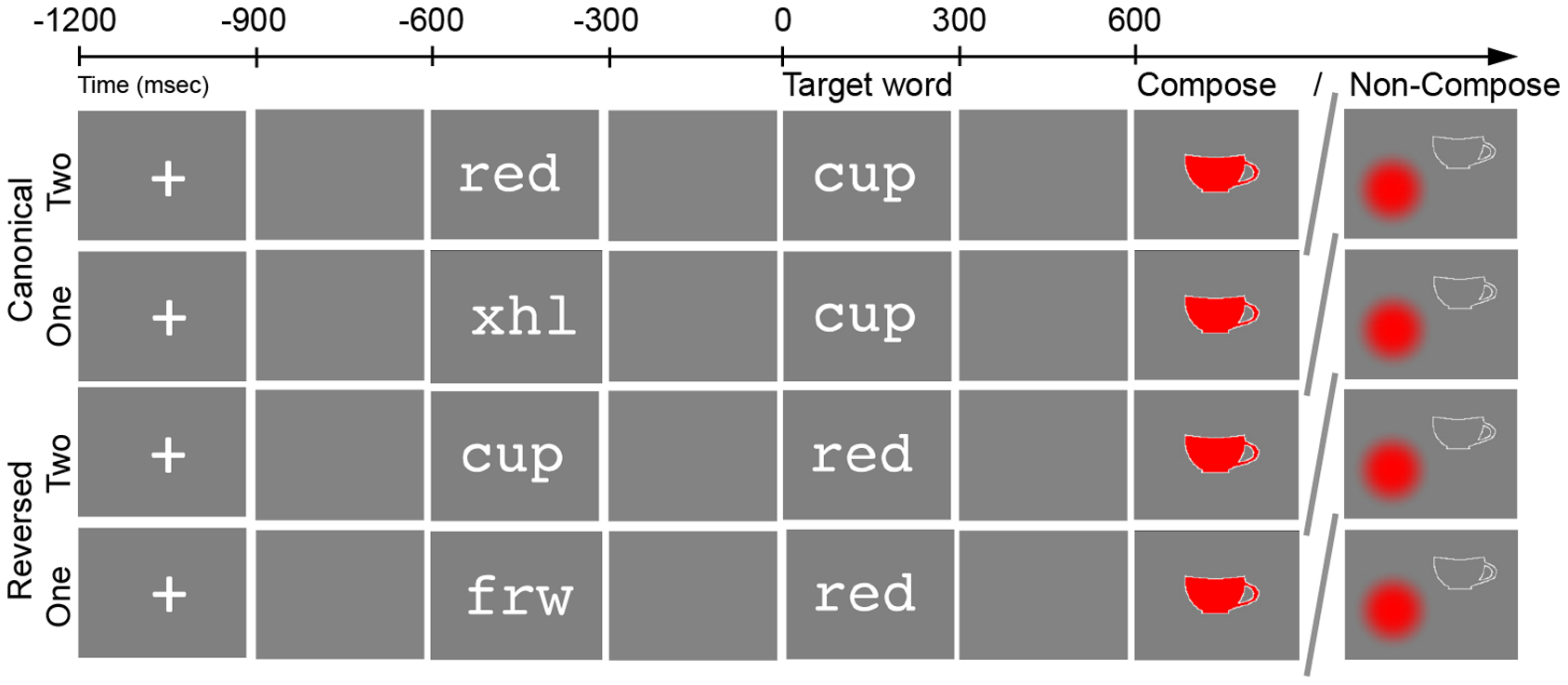
Experimental design. In each block of trials, subjects were presented with both one-word and two-word stimuli and asked to judge if a following target picture matched the preceding words. In the canonical conditions (top rows), stimuli were adjective-noun phrases (*red cup*) and their matched one-word controls (*xhl cup*). In the reversed conditions (bottom rows), stimuli were noun-adjective sequences (*cup red*) and their matched one-word controls (*frw red*). In the Compose task (left task picture), target pictures contained a single colored shape. In the Non-Compose task (right task picture), target pictures contained a colored blob and a shape outline. Each subject performed only one task and canonical and reversed trials were blocked separately.

In the present study, by contrast, we investigate whether basic combinatorial linguistic mechanisms can be rapidly deployed outside of their natural context within the native grammar and further, whether this deployment can be precipitated by a simple task manipulation independent of changes in complexity or attention. In other words, to what extent can basic combinatorial linguistic mechanisms be used as a flexible cognitive tool in solving tasks? Specifically, we investigated whether combinatorial mechanisms active during the composition of simple noun phrases can be flexibly used to interpret minimally contrasting ungrammatical sequences, if the task demands this. In our prior work we have characterized the combinatory neural activity elicited by the comprehension of simple adjective-noun combinations such *red cup*, finding reliable increased activity in the left anterior temporal lobe (LATL) at approximately 200 to 250 ms compared to non-combinatory controls [1], [2]. In the present study (Fig. 1), we contrasted such adjective-noun combinations with their reversed counterparts, *cup red*, which violate canonical English word order for such phrases. This stimulus manipulation was then embedded within a task manipulation that varied the necessity of combining the adjective and the noun into a single semantic representation. In the combinatorial (Compose) task, subjects judged whether the verbal stimulus matched a picture of a colored object. In the Non-Compose task, the task picture instead depicted a separate color blob and shape outline, allowing subjects to process the noun and adjective meanings in an entirely list-like fashion, with no composition. LATL activity hypothesized to reflect composition was measured by presenting each word sequentially and comparing the activity generated at the second word to that evoked during the processing of a matched non-combinatorial one-word control (*frw red* or *xtp cup*).

For the canonical word order stimuli, we expected combinatory processing to occur automatically in both tasks, given that nearly all parsing models hypothesize grammatical linguistic expressions to automatically engage combinatorial mechanisms to some degree, regardless of task (e.g. [50], [51]). This theoretical claim has been supported by numerous neurolinguistic investigations demonstrating that early electrophysiological components associated with combinatorial processing are invariant to both task [36], [37] and attentional manipulations [43], [33], [31]. Further, hemodynamic effects associated with combinatorial processing, including LATL effects driven by sentence processing, remain observable even during tasks specifically designed to minimize combinatorial processing [52], [53], [54].

During the processing of reversed order stimuli (*cup red*), in contrast, we did not expect combinatorial processing to be automatically evoked. Crucially we designed our tasks to require judgments about object denotations, thus deterring subjects from interpreting these sequences as modified colors (e.g. *wine red* is a particular shade of red associated with wine). Further, we selected word combinations that were not familiar noun-adjective colors (e.g. *beet red* was not used). It should be noted that within larger contexts adjectives may of course modify nouns post-nominally, e.g. *I saw a cup red with paint*, however, such usage generally requires the adjective to be sufficiently ‘heavy,’ and often requires an intonational break following the noun [55]. Thus, we expected that these reversed sequences should not engage combinatorial processing absent task pressure to do so. Consequently, we expected a contrast between the canonical and reversed sequences in terms of combinatorial processing during the Non-Compose task. Our primary question was then to determine the extent to which reversed sequences might engage combinatorial processing during the Compose task.

To keep the task manipulation as pure as possible, the two tasks were administered to separate groups of participants. Specifically, we wanted to avoid the possibility that after having processed the stimuli in a combinatory fashion, subjects might find it hard to disengage the combinatory mode of processing. In other words, such a task switching cost could lead to combinatory processing of the canonical word orders during the Non-Compose task not because composition is automatic for them but because of interference from a recently performed composition task.

In our previous work on grammatical adjective-noun sequences (*red cup*) [1], [2], the full activity pattern associated with combinatorial processing consisted of an early effect at *cup* localized to the LATL at approximately 200 to 250 ms followed by more variable effects later in the epoch (∼ 400 ms), localized to both the ventromedial prefrontal cortex [1]) and angular gyrus (AG - [2]). In the present study, we chose to use MEG activity localized to the LATL as our primary index of basic combinatorial processing for two reasons. First, a rather expansive hemodynamic literature suggests that this region is crucially involved in combinatorial linguistic processing [56], [57], [58], [59], [54], and both previous versions of this paradigm, across visual and auditory presentations, have robustly produced combinatorial activity localized to the LATL [1], [2]. Later effects have been more variable. Second, as discussed above, later processing components, and specifically those elicited during the time window surrounding the previously observed vmPFC and AG effects (350 - 450 ms), can be heavily influenced by task manipulations independent of any variation in combinatorial processing (see [41]). Thus, earlier components are better candidates for measures that might more directly reflect combinatorial processing when manipulating tasks.

To summarize, if our assumptions are correct and canonical adjective-noun combinations do engage combinatorial mechanisms regardless of task demands, but reversed noun-adjective sequences do not, then we expect to see LATL activity in the Non-Compose task exhibit an interaction between the reversed (*cup red*) and canonical sequences (*red cup*), compared to their matched one-word controls (*xhl red* and *frw cup*, respectively), with increased LATL activity only present during the two-word canonical condition. If the Compose task then causes basic combinatorial mechanisms to be flexibly engaged during the processing of reverse sequences, we expect to see a main effect of number of words in this task, with increased LATL activity present in both two-word sequences relative to their matched controls. On the other hand, if the task manipulation is not sufficient to evoke combinatorial processing in the reversed sequences, then we would again expect to observe an interaction between order and number of words in this task as well. To maximize the strength of the manipulation, we administered each task separately to different subjects.

## Methods

### Participants

15 subjects performed the Non-Compose task (8 female; mean age of 22.4 years). 21 subjects performed the Compose task (14 female; mean age of 21.4 years). All subjects were right-handed, non-colorblind native English speakers with normal or corrected-to-normal vision. All procedures were approved by New York University’s Committee on Activities Involving Human Subjects and informed written consent was obtained from each participant. Participants received a fee or course credit for their participation.

### Materials

Each trial contained four stimuli that were presented sequentially: a fixation cross, an initial word or non-word, a noun or adjective, and a target picture (Figure 1). Subjects were told to ignore all non-word stimuli and indicate if the target picture contained a depiction of all of the preceding lexical items. In the Compose task, the target picture contained a single colored shape. In the Non-Compose task, the color and shape were presented separately as a circular blob and a white outline, respectively. Linguistic stimuli varied by condition: two-word canonical trials presented an adjective followed by a noun (*red cup*), two-word reverse trials presented a noun followed by an adjective (*cup red*), and one-word trials replaced initial words with unpronounceable consonant strings (*xhl cup, frw red*). Thus, the second, critical stimulus remained unchanged between paired one and two-word conditions. During the Non-Compose experiment subjects also completed a list variant of each task, in which they judged whether the target picture matched either of two preceding nouns or adjectives. As this contrast is not relevant to the hypotheses of the present study, it has been omitted from the following discussion.

Throughout all conditions, nine one-syllable, common color adjectives were used (*red, tan, teal, blue, pink, black, brown, white, green*). Each adjective was assigned a corresponding length-match noun (*cup, car, lock, shoe, leaf, house, heart, plane, cross*) and a corresponding length-match unpronounceable consonant string (*xkq, kjw, qxsw, mtpv, vbnw, rjdnw, wvcnz, zbxlv, vtzkn*). Nouns and adjectives were also matched for frequency (*p* = 0.74; HAL log frequency; paired *t*-test). Each word was displayed in Courier non-proportional font and subtended approximately 3°. In the Compose task, target pictures were hand-created canonical depictions of each shape, colored in with one of the nine colors and displayed in the center of the screen, subtending approximately 8°. In the Non-Compose task, circular blobs and outlines (taken from the colored shapes) were randomly placed at one of four locations, centered +/−2° both horizontally and vertically from the center of the screen, with no two objects occupying the same location on any trial. For these stimuli, each object subtended approximately 4° on its own. All stimuli were presented using psychtoolbox [60], [61] and projected onto a screen approximately 45 cm from the subject’s eye.

In each task, canonical and reversed trials were blocked separately, with order counter-balanced across each group of subjects. In each condition, critical items (i.e. nouns for canonical trials, adjective for reversed trials) were presented four times in matching trials and four times in non-matching trials, resulting in a total of 72 trials in each condition and 144 trials per block. In two-word non-matching trials, target pictures matched either the preceding color or shape term but not both. For each subject, two-word reverse trials were created by simply reversing the order of the stimuli in the two-word canonical trials. One-word trials were then created from all two-word trials by substituting a matched consonant string for each initial stimulus and shuffling the target pictures to match or not match the remaining word as needed. Stimuli lists were randomized per subject.

### Procedure

Before the experiment, subjects practiced their first block outside of the MEG room. Prior to recording, subjects’ head shapes were digitized using a Polhemus Fastrak 3D digitizer (Polhemus, VT, USA). The digitized head shape was then used to constrain source localization during analysis by co-registering five coils located around the face with respect to the MEG sensors. Additionally, electrodes were attached 1 cm to the right of and 1 cm beneath the middle of the right eye in order to record the vertical and horizontal electrooculogram (EOG) and detect blinks. Both electrodes were referenced to the left mastoid.

MEG data were collected using a using a whole-head 157-channel axial gradiometer system (Kanazawa Institute of Technology, Tokyo, Japan) sampling at 1000 Hz with a low-pass filter at 200 Hz and a notch filter at 60 Hz. All stimuli besides the target pictures were presented for 300 ms, followed by a 300 ms blank screen. Target pictures appeared at the end of each trial and remained onscreen until the subject made a decision. Subsequent trials began after a blank screen was shown for a variable amount of time following a normal distribution with a mean of 500 ms and a standard deviation of 100 ms (see Figure 1). The entire recording session lasted approximately 50 minutes for the Non-Compose task and 25 minutes for the Compose task (difference being due to the list blocks included in the Non-Compose task; see Materials above).

### MEG Data Acquisition

MEG data was recorded continuously, noise-reduced using the Continuously Adjusted Least- Squares Method (CALM; [62]) and epoched from 100 ms prior to the onset of each critical item to 600 ms post onset. Raw data were first cleaned of potential artifacts by rejecting trials for which: a) the subject answered either incorrectly or too slowly (defined as more than 2.5 seconds after the appearance of the target shape), b) the maximum amplitude exceeded 3000 fT, or c) the subject blinked within the critical time window, as determined by manual inspection of the EOG recordings. One subject in the Non-Compose task performed at chance in the one-word reverse condition and was therefore excluded from further analysis. Remaining data were then averaged for each subject for each condition and band-pass filtered between 1 and 40 Hz. For inclusion in further analysis, we required that subjects show a qualitatively canonical profile of evoked responses during the processing of the critical items. This profile was defined as the appearance of robust and prominent initial visual responses – i.e. either the M100 or M170 field pattern [63], [64] had to be clearly present in the time window of 100 to 200 ms following the critical stimuli. Five subjects overall failed to meet this requirement (three in the Compose task and two in the Non-Compose task) and were excluded from further analysis.

### Minimum Norm Estimates

As our primary dependent measure, we created distributed minimum norm source estimates for recorded MEG sensor data. This measure provides an estimate of the cortical location of electrical activity underlying the observed magnetic fields recorded outside of the head. A source estimate was constructed for each condition average using L2 minimum norm estimates calculated using BESA 5.1 (MEGIS Software GmbH, Munich, Germany). The channel noise covariance matrix for each estimate was based upon the 100 ms prior to the onset of the critical item in each condition average. Each minimum norm estimate was based on the activity of 1426 regional sources evenly distributed in two shells 10% and 30% below a smoothed standard brain surface. Regional sources in MEG can be regarded as sources with two single dipoles at the same location but with orthogonal orientations. The total activity of each regional source was then computed as the root mean square of the source activities of its two components. Pairs of dipoles at each location were first averaged and then the larger value from each source pair was chosen, creating 713 non-directional sources for which activation could be compared across subjects and conditions. Minimum norm images were depth weighted as well as spatio-temporally weighted using a signal subspace correlation measure [65]. Repeated measures analysis of variance within each task with the factors Order (canonical, reversed) and Number of words (two, one) revealed no reliable main effects or interactions in the signal-to-noise ratio of the minimum norm estimates (all p’s >.15).

### Data Analysis

To assess combinatorial activity, our primary analysis examined source activity localized to the LATL, as defined by the ROI used in our previous experiments [1], [2]. The boundary of this ROI was initially based upon hemodynamic results demonstrating increased activity in the LATL during the comprehension of sentences compared to word lists [57], [56], [54]. We analyzed activity that localized to this ROI from 0 to 600 ms following the presentation of the second word using a cluster-based permutation test [66] designed to identify significant effects at any point during the analysis epoch. This test controls for multiple comparisons within the entire time interval using the following generalized procedure. First, a cluster test statistic is calculated for the observed data. Then, this same test statistic is calculated for many permutations of the actual data, each created by randomly shuffling the condition labels within each participant. The *p* value of the observed test statistic is then computed relative to a distribution created from 10,000 permutations of the original data and is set equal to the proportion of permuted datasets that produce a test statistic more extreme than that of the actual data.

One benefit of this test is that the cluster test statistic can be constructed specifically in order to match a particular hypothesis (see [1]; [66]). In the present experiment, each statistic was calculated by first identifying contiguous time points for which a statistical point-wise test reached a certain threshold (set to *p = *0.30 to match our previous analyses) and then calculating a single test statistic from the resulting cluster of points. To test for the interaction predicted in the non-combinatorial task, we used the same test statistic as in our previous paper (see [1] for full details). Thus, each cluster was identified using the interaction *p* value from the point-wise 2×2 repeated-measures ANOVAs, with number of words (one, two) and word order (canonical, reversed) as factors. The final test statistic was then calculated by summing the *t* values resulting from the point-wise *t* tests between the canonical conditions and subtracting the *t* values resulting from the point-wise *t* tests between the reversed conditions. To test for the main effect predicted in the combinatorial task, we identified clusters using the *p* value from the point-wise 2×2 repeated-measures ANOVA that corresponded to the main effect of number of words, and we included only those points for which the interaction *p* value was not also above threshold. Then, we simply summed the *t* statistics from all *t* tests performed within the two condition pairs to calculate the final test statistic.

To identify effects outside of the LATL, we also performed a full-brain analysis that compared two-word and one-word activity measures within each task, sample by sample for every source-time point using a paired *t* test. A difference was considered significant in this test only if it surpassed a significance and size criteria such that it remained reliable (*p*<0.05) for at least 10 samples (10 ms), was observed in at least 10 adjacent cortical sources, and was least 1.5 nAm in amplitude. In the results and figures below, we discuss only effects attributable to an increase in two-word activity.

## Results

### Behavioral Results

**Figure 2:**
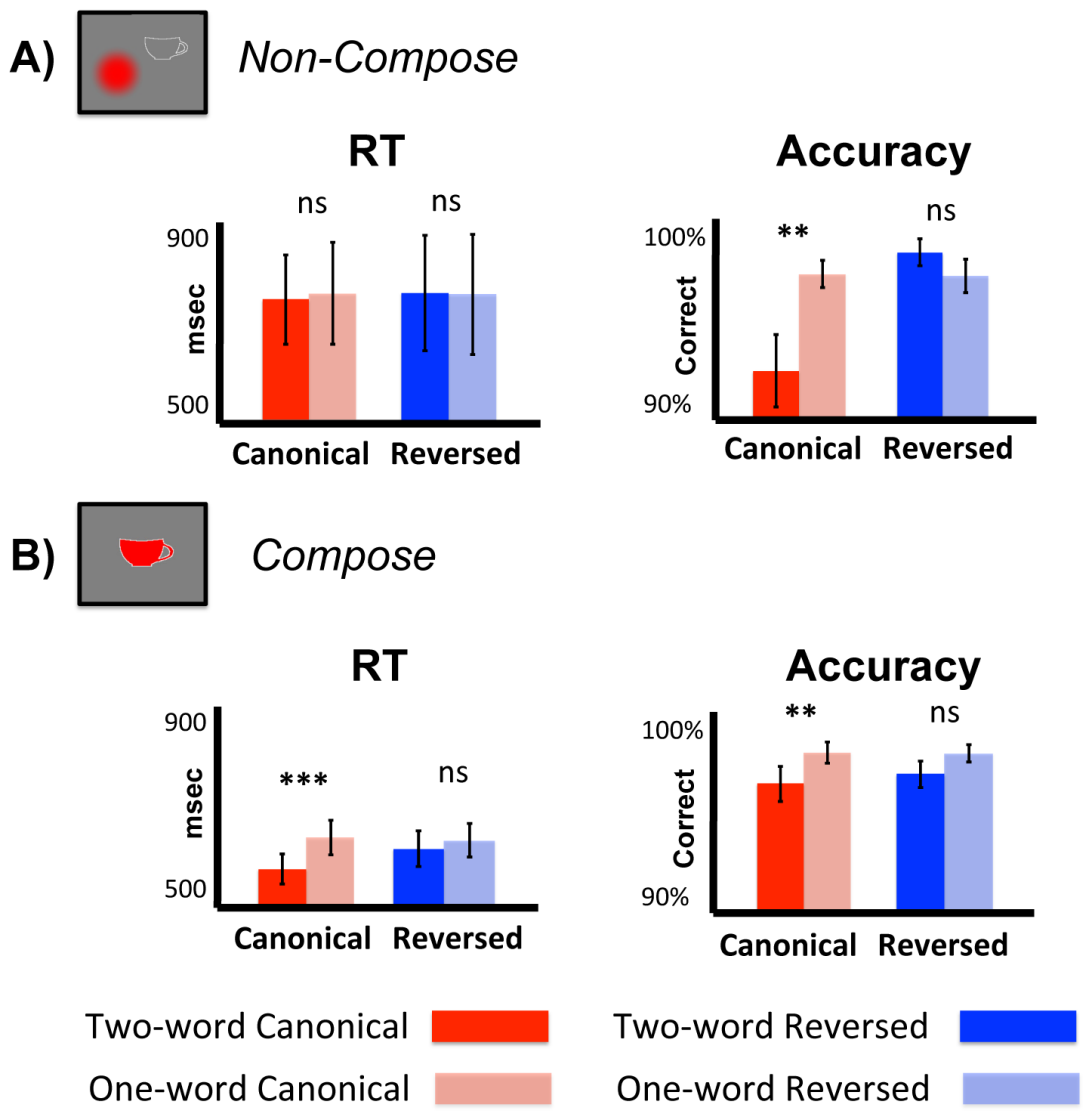
Behavioral results. For each task, reaction time and accuracy data were submitted to a 2×2 repeated-measures ANOVA with order (canonical, reversed) and number of words (one, two) as factors. In the Non-Compose task (A), we observed a significant interaction between the two factors for accuracy and found no significant effects for reaction time. In the Compose task (B), we observed a significant main effect of number of words for accuracy and a significant interaction between the two factors for reaction time. ns, Nonsignificant; ***p<0.001; **p<0.01.

Behavioral data, depicted in Fig. 2, were analyzed for speed and accuracy in a 2×2×2 ANOVA with Task (Compose, Non-Compose) as a between subjects variable and Order (canonical, reversed)×Number of words (two, one) as within subjects variables. This was followed by planned repeated-measures 2×2 ANOVAs on the effects of Order and Number of words within each task.

For accuracy, the 2×2×2 ANOVA revealed a significant three-way interaction between task (Compose, Non-Compose), order (canonical, reversed), and number of words (two, one) (*F*(1,28) = 6.62; *p* = 0.016). The subsequent 2×2 ANOVA within the Non-compose task showed a significant interaction between order and number of words for accuracy (*F*(1,11) = 9.03, *p* = 0.012), with follow-up paired *t*-tests between one- and two-word condition pairs revealed that this effect was driven by significantly lower accuracy in the two-word canonical condition compared to the matched one-word control (*p = *0.007; two-words: 91.9% avg. [1.8% std.]; one-word: 96.8% avg. [0.7% std.]), and no statistical difference in accuracy for the reversed order conditions (*p* = 0.39; two-words: 97.9% avg. [0.8% std.]; one-word: 97.2% avg. [0.7% std.]). The corresponding 2×2 ANOVA within the Compose task, on the other hand produced no main effect of order or interaction between the two factors (both *F*s <1), but a significant main effect of number of words (*F*(1,17) = 13.94; *p* = 0.0017), with lower accuracy in both two-word conditions compared to their matched controls (canonical two-words: 96.4% avg. [0.8% std.]; canonical one-word: 98.1% avg. [0.5% std.]; reversed two-words: 97.1% avg. [0.4% std.]; reversed one-word: 98.0% avg. [0.4% std.]). Thus the interaction observed in the initial 2×2×2 ANOVA was driven by a significant effect of Task on the interaction between number of words and order in accuracy.

For reaction time, the 2×2×2 ANOVA showed a main effect of Task (F(1,28) = 6.96; *p*<0.01), with responses faster in the Compose task compared to the Non-Compose task (622 ms avg. [139 ms std.] v. 745 ms avg. [349 ms std.]). There was no significant three-way interaction (*F*(1,28) = 1.88, *p* = 0.18) though the eight conditions again displayed the same qualitative pattern as for accuracy, with every two-word condition differing from its paired one-word control in the same manner (here, with faster responses) except for the two-word reversed condition in the Non-Compose task, which did not differ from its matched one-word control (and in fact had slightly slower responses). The 2×2 ANOVA within the Non-Compose task showed no significant effects in reaction times for order, number of words, or their interaction (all *F* values <1; canonical: two-words: 727 ms avg. [86 ms std.]; one-word: 739 ms avg. [98 ms std.]; reversed: two-words: 759 ms avg. [113 ms std.]; one-word: 757 ms avg. [116 ms std.]). The corresponding 2×2 ANOVA within the Compose task did however show a significant interaction between order and number of words (*F*(1,17) = 8.39; *p* = 0.01). Follow-up paired *t*-tests revealed that this effect was driven by significantly faster responses in the two-word canonical condition compared to the matched control (*p*<0.001; two-words: 579 ms avg. [30 ms std.]; one-word: 644 ms avg. [38 ms std.]) and no statistical difference between the reversed order conditions (*p = *0.40; two-words: 625 ms avg. [36 ms std.]; one-word: 640 ms avg. [33 ms std.]).

Thus, overall, our behavioral measures suggest a clear dissociation between all of the two-word conditions and their one-word controls, except for the two-word reversed condition in the Non-Compose task, which instead closely matched its one-word control. This results pattern, of course, is precisely that predicted for combinatorial processing if combinatorial mechanisms can be flexibly deployed in the reversed two-word sequences when the task demands composition and not automatically engaged when the task does not. Further, the faster response times exhibited by the composed two-word conditions echoes the facilitation effect previously observed for composed phrases in this task (Bemis & Pylkkänen, 2011). While the cause of the accompanying decrease in accuracy for these conditions is unclear, and suggests a previously unobserved speed-accuracy trade-off, in general the behavioral results suggest that processing was similar during canonical and reversed two-word sequences when the task demanded composition and dissimilar when no composition was required.

### Left Anterior Temporal Lobe ROI Results

#### Non-Compose LATL results

**Figure 3:**
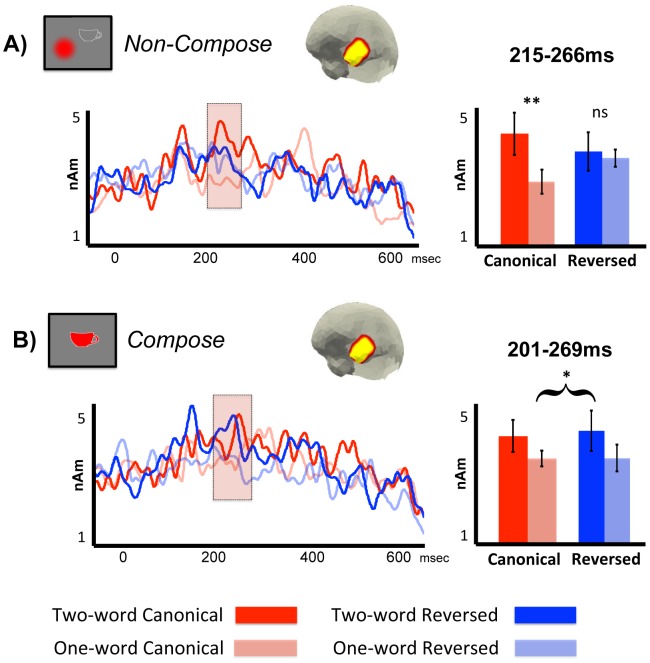
LATL ROI results. Localized activity is shown for the LATL ROI during the processing of the critical items (nouns in canonical sequences, adjectives in reversed sequences), averaged across subjects. Shaded regions denote significant clusters of combinatorial activity, as determined by a non-parametric, cluster-based permutation test (Maris & Oostenveld, 2007) applied to the entire epoch, from 0 to 600 ms following the presentation of the critical item. We observed a significant interaction between order and number of words in the Non-Compose task (A) and a significant main effect of number of words in the Compose task (B) for activity generated between 200 and 300 ms. ns, Nonsignificant; **p<0.01; *p<0.05.

In the Non-Compose task, the interaction permutation test identified a significant cluster of activity localized to the LATL (Figure 3) from 215 to 266 ms (*p* = 0.023; 10,000 permutations). A 2×2 repeated measures ANOVA performed on LATL activity averaged across this time window supported this result and demonstrated a significant interaction between order and number of words (*F*(1,11) = 7.361; *p* = 0.020) with activity in the two-word canonical condition significantly greater than in the matched one-word control (*p* = 0.009; paired samples *t* test; two-word: 4.25 nAm avg. [2.14 nAm std.]; one-word: 2.86 nAm avg. [1.23 nAm std.]), and no statistical difference between activity in the reversed conditions (*p* = 0.732; paired samples *t* test; two-word: 3.70 nAm avg. [1.95 nAm std.]; one-word: 3.51 nAm avg. [0.86 nAm std.]).

We found no evidence of increased LATL activity during the processing of reversed sequences in this task. Both the main effect permutation test and a *post-hoc* cluster test performed between the two reversed conditions alone failed to find any significant clusters of activity (all clusters *p*>0.8). Thus, in the Non-Compose task we observed a significant combinatorial effect during the processing of canonical phrases at a time similar to that observed in our previous experiments (Bemis & Pylkkänen, 2011, 2012) and no evidence of any combinatorial LATL effects during the processing of the reversed sequences at any time.

#### Compose LATL results

In the Compose task, the permutation test designed to identify increased activity in both two-word conditions identified a significant cluster of activity localized to the LATL (Figure 3) from 201 to 269 ms (*p* = 0.029; 10,000 permutations). A 2×2 repeated-measures ANOVA performed on activity averaged across this cluster supported this result and identified a significant main effect of number of words (*F*(1,17) = 4.52; *p* = 0.0385) with increased activity in both two-word conditions compared to their paired one-word controls (two-word canonical: 4.00 nAm avg. [1.93 nAm std.]; one-word canonical: 3.37 nAm avg. [0.97 nAm std.]; two-word reversed: 4.17 nAm avg. [2.43 nAm std.]; one-word reversed: 3.39 nAm avg. [1.61 nAm std.]). Neither the main effect of order nor the interaction between the two factors was significant in this test (both *F*s <1).

This permutation test also identified a marginally significant cluster of activity from 380 to 430 ms (*p* = 0.075; 10,000 permutations), corroborated by a marginal main effect of number of words according to a 2×2 repeated-measures ANOVA on activity during this time window (*F*(1,17) = 3.52; *p* = 0.078; two-word canonical: 3.72 nAm avg. [1.96 nAm std.]; one-word canonical: 3.13 nAm avg. [1.20 nAm std.]; two-word reversed: 3.80 nAm avg. [2.32 nAm std.]; one-word reversed: 2.95 nAm avg. [1.33 nAm std.]). Again, there was no effect of order in this time window and no interaction between the two factors (both *F*s <1). This result again closely matches our previous findings, in which we observed a marginally significant increase in combinatorial activity in the LATL from approximately 350 to 400 ms (Bemis & Pylkkänen, 2011).

The only effect identified by the interaction permutation test in this task was a marginally significant cluster of activity from 469 to 505 ms (*p* = 0.079; 10,000 permutations) in which LATL activity increased during the two-word canonical condition only (two-word canonical: 3.77 nAm avg. [1.43 nAm std.]; one-word canonical: 2.70 nAm avg. [0.87 nAm std.]; two-word reversed: 2.72 nAm avg. [2.12 nAm std.]; one-word reversed: 2.83 nAm avg. [1.52 nAm std.]). This interaction was significant according to a 2×2 repeated-measures ANOVA on activity during this time window (*F*(1,17) = 6.79; *p* = 0.019). No other significant effects were identified at any point in the analysis epoch using any test.

Thus, in general, the results from our LATL ROI analysis strongly indicate a grouping of canonical and reversed sequence processing during the Compose task and a dissociation between the two during the Non-Compose task.

### Full-brain Results

#### Non-Compose full-brain results

**Figure 4:**
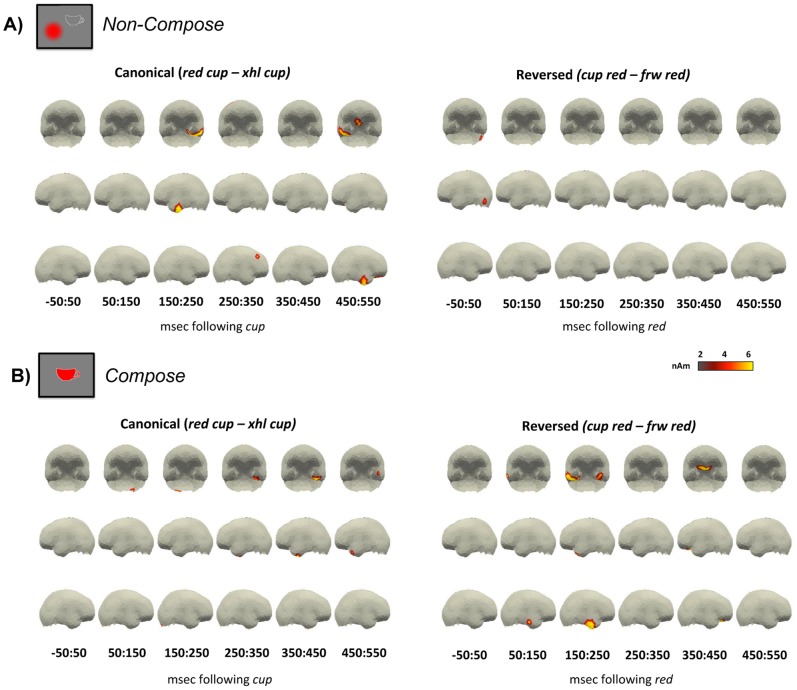
Full-brain results. Plotted regions denote the difference in average amplitude between two-word and one-word conditions for all space time regions in which two-word activity was reliably greater than one-word activity (*p*<0.05, uncorrected) for at least 10 ms over 10 spatial neighbors, and the amplitude of the differences was at least 1.5 nAm. For clarity, non-cortical sources have been removed. In general, the results within both the Non-Compose (A) and Compose (B) task conform to our ROI analysis, revealing clear LATL effects in the earlier time window, at approximately 200 to 300 ms. Later effects are also visible in the RATL and vmPFC for both canonical phrases in the Non-Compose task and reversed sequences in the Compose task.

For the canonical ordering, a clear increase in activity can be seen (Figure 4) from 150 to 250 ms in the LATL, thus supporting the ROI analysis above. Additional effects are also visible in the ventromedial prefrontal cortex (vmPFC) and right anterior temporal lobe (RATL) from 450 to 550 ms. Increased activity in both of these regions was also observed during the processing of adjective-noun phrases in our previous study, though both effects began slightly earlier in those results and only activity in the vmPFC appeared to reflect combinatorial processing, with RATL activity seemingly dependent upon the task (see [1] for details).

Within the reversed sequences, we observed very few increases in activity during two-word processing, further supporting the LATL ROI analysis and behavioral results in suggesting that processing in the two-word reverse condition did not differ from the matched one-word control in this task.

#### Compose full-brain results

In the Compose task, a clear increase in LATL activity can be observed (Figure 4) during the processing of two-word canonical phrases from 250 to 550 ms. This conforms closely to our ROI analysis, which identified increased activity in this region throughout the later half of the analysis epoch. No other effects of note are visible within this comparison.

For the reversed expressions, the full-brain comparison again conformed to our ROI analysis, revealing a clear increase in LATL activity from 150 to 250 ms. Outside of this ROI, effects were again clearly visible in the RATL, concurrent with the LATL effect, and in the vmPFC, following the LATL effect. As mentioned before, this pattern of activity aligns very closely to our previous findings for canonical adjective-noun phrases within the same paradigm [1].

## Discussion

In the present study, we investigated whether basic combinatorial linguistic processing can be flexibly deployed to novel expressions given only a minimal change in task demands. We recorded MEG activity as subjects read simple adjective-noun sequences in canonical or reversed order (*red cup*, *cup red*) and measured combinatorial processing by comparing activity evoked by the presentation of the second word with that evoked during the processing of matched, non-combinatorial controls (*xhl cup*, *frw red*). We used neural activity localized to the LATL during the processing of the matched critical words as our primary measure of combinatorial activity. Previous MEG investigations using a similar paradigm have observed consistent and robust combinatorial effects in this region during the comprehension of simple adjective-noun phrases [1], [2], and many hemodynamic investigations have observed increased activity in the LATL during the comprehension of sentences compared to word lists [57], [59], [54] In the present study, when subjects were required to judge whether the given linguistic stimuli matched a following colored blob and shape outline, no combinatorial LATL activity was observed for reversed sequences but was robustly present for canonical phrases. When the following target was instead presented as a single colored shape, significant combinatorial activity was observed for both sequence types. This pattern of results was echoed in our behavioral measures, which demonstrated consistent differences between all two-word and one-word responses with the exception of two-word reversed sequences in the Non-Compose task, which patterned instead with their one-word controls. Thus, the present data, both behavioral and neural, indicate that reversed noun-adjective sequences were processed similarly to canonical adjective-noun phrases when the task demanded composition and similarly to non-combinatorial controls when it did not.

The importance of these results is two-fold. First, the robust LATL activity observed during the canonical phrases in the Non-Compose task further validates this measure as an index of basic combinatorial processing. Many previous investigations into the automaticity of linguistic parsing mechanisms indicate that hemodynamic combinatorial effects localized to the LATL [54] and early electrophysiological components associated with the parsing of grammatical expressions [37], [33] remain robustly present even during explicitly non-combinatorial tasks. Thus, if early activity localized to the LATL in the present paradigm does indeed reflect basic combinatorial processing, it would be expected to exhibit the observed profile and remain robust during our task manipulation. Second, the identification of this effect during the processing of reversed sequences in the Compose task, and not in the Non-Compose task, indicates that the combinatorial linguistic mechanisms indexed by this measure can be flexibly engaged outside of their normal context given only a minimal change in task demands. Interestingly, the observed effect in the reversed sequences coincided temporally with that in the canonical phrases (both occurred at approximately 200 to 250 ms) suggesting a similar time-course for this combinatorial mechanism across sequence types. Though potentially counter-intuitive, this apparent ease in extending combinatorial processing to a novel context may have been facilitated by the blocked nature of the present paradigm. Future work might indicate that a more unexpected shift in task demands results in a more delayed engagement of combinatorial processing.

Past investigations into the effects of task demands on language processing have focused nearly exclusively on manipulations of attention, either explicitly [33] or implicitly [32]. Even the few studies that have employed tasks potentially able to address the flexibility of combinatorial processing (e.g. [67] ) have instead remained explicitly focused on measuring the effect of attention. In direct contrast, the present study sought to minimize changes in attention while manipulating only the combinatorial processing required. In both of our tasks, subjects had to retrieve the semantic representation of all words in order to determine whether the target picture contained their denotation. Thus, processing related to lexical access and attention should be relatively equivalent between the two tasks. It is therefore difficult to attribute the increase in LATL activity observed during the processing of reversed sequences in the Compose task to changes in attention, especially as the same activity was robustly present for canonical phrases during the Non-Compose task as well. Thus, unlike past investigations, the present study demonstrates the flexible engagement of combinatorial linguistic mechanisms evoked by a change in task demands that does not involve an explicit manipulation of attention.

### Later Processing Components

We found additional evidence for the flexible engagement of combinatorial processing outside of early LATL activity as well. During the processing of reversed sequences in the Compose task, we observed a marginally significant increase in LATL activity from approximately 350 to 400 ms and additional combinatorial activity localized to the vmPFC from 400 to 500 ms – a pattern of effects strikingly similar to that previously observed for canonical phrases [1]. No such effects were observed in the Non-Compose task. Thus, results from later processing also support the conclusion that basic combinatorial mechanisms were engaged during the processing of reversed sequences in the Compose task.

This evidence, however, is not quite as strong as for the earlier LATL effects, as these later results were not as robustly replicated in the present experiment for the canonical phrases. While we did observe increased activity localized to the vmPFC during the processing of canonical phrases in the Non-Compose task from approximately 450 to 550 ms, this effect was not replicated in the Compose task as well. Instead, combinatorial activity remained localized to the LATL for the remainder of the epoch in this task and did not migrate to other regions, such as either the vmPFC [1] or the AG [2]. This deviant result was especially unexpected given that this particular contrast did not differ in any structural way from that used in our previous studies. One possible explanation for the observed difference might be that the present study used only nine critical items whereas both previous studies employed at least 20. This decrease in lexical variability might have contributed to the relatively muted and variable nature of later effects in this contrast, as decreased lexical variability has often been associated with decreased neural activity (e.g. [68]). However, the robust nature of later effects observed during the reversed sequences in this task suggests that this explanation is incomplete. More work is clearly needed in order to untangle the various mechanisms that underlie this later processing stage, especially given the variability observed for such effects in our previous results as well.

### LATL Function

While the present results speak to *how* the combinatory mechanism housed in the LATL operates, our findings are compatible with several functional hypotheses of exactly *what* this mechanism computes. Even the most basic grammatical processing is hypothesized to involve both syntactic and semantic mechanisms, responsible for forming structural relationships and constructing complex meanings from individual elements. Previously [1], [2], we have suggested a tentative mapping of syntactic and semantic processes onto the early LATL and late vmPFC effects respectively. This suggestion was based upon previous work linking hemodynamic activity in the LATL to combinatorial syntactic processing, [69], [70], MEG work linking increased vmPFC activity to semantic composition [71], [72], and neurolinguistic processing models that posit syntactic combination prior to semantic composition [73]. On one view, the present results might be seen as supporting this proposed delineation, as many past studies associate early, automatic electrophysiological components with syntactic processes [36], [32], [33] and later, more task-dependent components with semantic processing [41], [40]. Thus, our finding that early combinatorial activity in the LATL remains robust across tasks during the processing of canonical phrases might support the suggestion that this component reflects syntactic processing.

On the other hand, task-robust early electrophysiological components have also been attributed to semantic processing [74], and recent hemodynamic work argues that increased activity in the LATL during the comprehension of sentences reflects semantic, and not syntactic, combinatorial processing [59], [35]. In the present study, the nature of the task manipulation also suggests a semantic, and not syntactic, role for the LATL, as the only difference between the two tasks is that in the Compose task subjects must construct a singular semantic representation from the individual shape and color representations. No obvious syntactic manipulation is involved, as the linguistic stimuli remain constant between tasks. Thus, it is difficult to provide a simple functional explanation for how, or for that matter why, a syntactic phrase might be formed for the reversed sequences in this task. It certainly may be the case that the parser forms such a constituent despite the reversed order of the constituents – and some have argued that this must be the case in order for semantic composition to proceed (e.g. [36]) – however, it seems more plausible that the combinatorial activity observed during the Compose task simply reflects the demands of the task, i.e. the semantic composition of the two elements. More specifically, this activity may reflect a type of specification process in which the basic conceptual representation of the object is transformed into a more complex form that represents the more specific concept of a colored shape. Such an operation could, arguably, proceed regardless of the order in which the elements are encountered, and such symmetry is in fact a formal property of theoretical accounts of modification [75]. Further, several previous studies have associated activity in the LATL with precisely this type of specification operation, though during the processing of single words [76], [77]. If this hypothesis is correct then later combinatorial effects might constitute a second stage of semantic composition, matching several models of conceptual modification based upon psycholinguistic results (e.g. [78], [79]. For the time being, however, this conclusion must remain tentative, as the present manipulation was not specifically designed to disentangle these functional hypotheses.

### Conclusion

In the present study, we demonstrate that basic combinatorial linguistic mechanisms can be flexibly deployed to a context beyond their natural grammatical domain. In contrast to previous studies that manipulate attention or utilize lengthy implicit learning paradigms, we show here that merely introducing the relevance of combination, while holding attentional and lexical factors constant, is sufficient to elicit basic combinatorial processing between two lexical items that do not naturally engage such processing. Future work may now build on this result in order to determine the extent of this flexibility both in terms of linguistic mechanisms and cognitive domains.
